# Surgical management of a left anterior descending Coronary Artery Aneurysm after drug eluting stent implantation

**DOI:** 10.1016/j.ijcha.2021.100793

**Published:** 2021-05-08

**Authors:** Yavuzer Koza, Oğuzhan Birdal, Hakan Taş, Noorullah Hamdard, Ferhat Borulu, Bilgehan Erkut

**Affiliations:** Ataturk University, Faculty of Medicine, Department of Cardiology, Erzurum 25100, Turkey; Cardiovascular Surgery, Erzurum 25100, Turkey

Coronary artery aneurysm (CAA) formation after percutaneous coronary intervention (PCI) is a rare complication with an incidence of 0.3–6.0% [1]. Most of them are pseudoaneurysms rather than true aneurysms. The clinical presentation of these aneurysms is widely variable as some patients are asymptomatic, but some have complaints of angina or present with acute coronary syndrome (ACS) [2], [3]. Herein, we report a rare case of CAA developed after PCI with a drug eluting stent (DES) who presented with ACS.

A 38-year-old man with a past history of anterior wall myocardial infarction for which stenting of the left anterior descending (LAD) artery with a Promus Element (Boston scientific, USA) stent 3 × 28 mm at 12 atm with post dilatation at 20 atm using a non-compliant balloon (Fig. 1A/B) presented with ACS 10 months later after stenting. Coronary angiography showed an aneurysm extending to the left main coronary artery at the site of previous stent deployment (Fig. 2A/B). Because aneurysm developed in a previously stented segment with a significant involvement of the left main coronary artery, we decided to perform surgery. The patient underwent on-pump coronary artery bypass surgery with a left internal mammarian artery graft to the proximal LAD artery and a saphenous vein graft to the left circumflex artery. The aneurysm was proximally ligated and plicated (Fig. 2C). The postoperative course was uneventful and he was discharged 11 days after the operation.

**Fig. 1:**
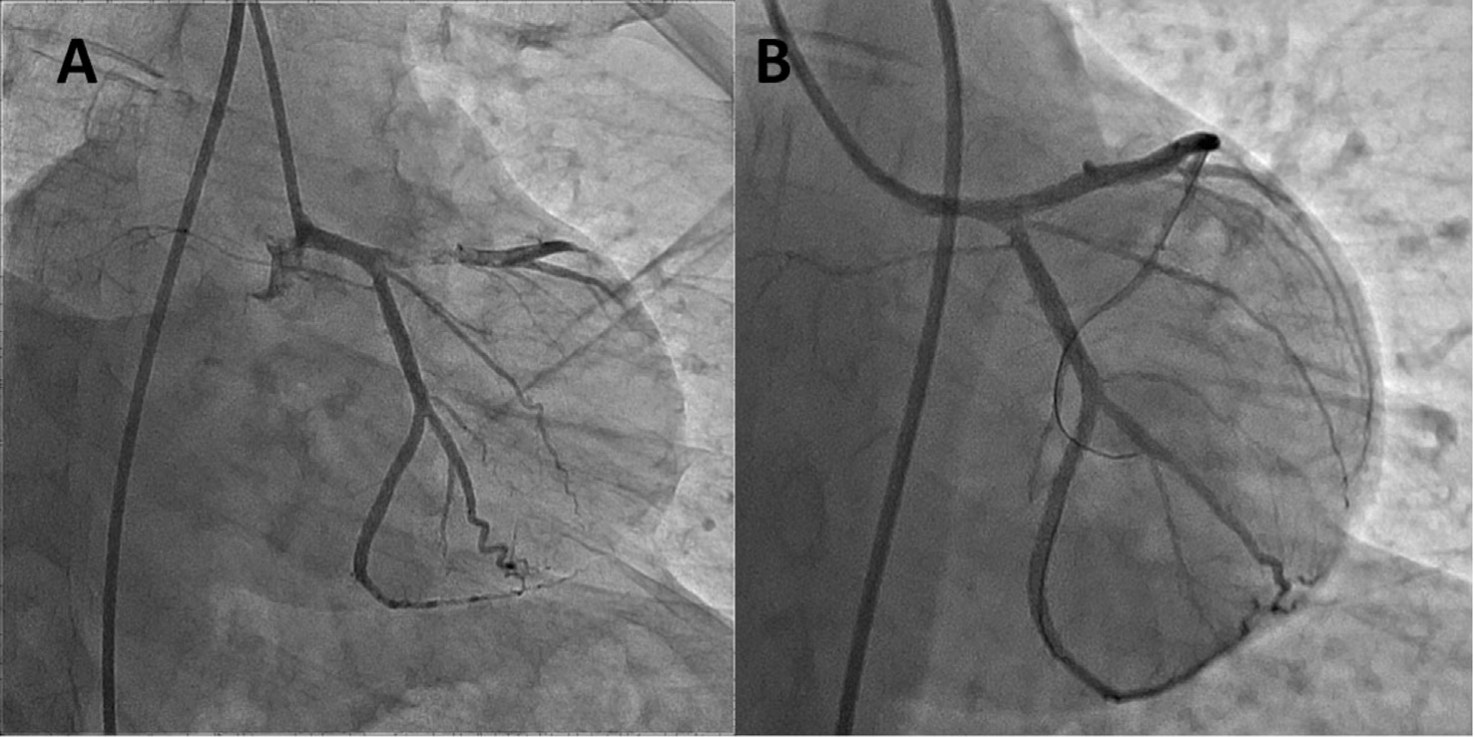
Angiographic image showing the proximal left anterior descending artery lesion with a high thrombus burden (A), final result after stent deployment (B).

**Fig. 2:**
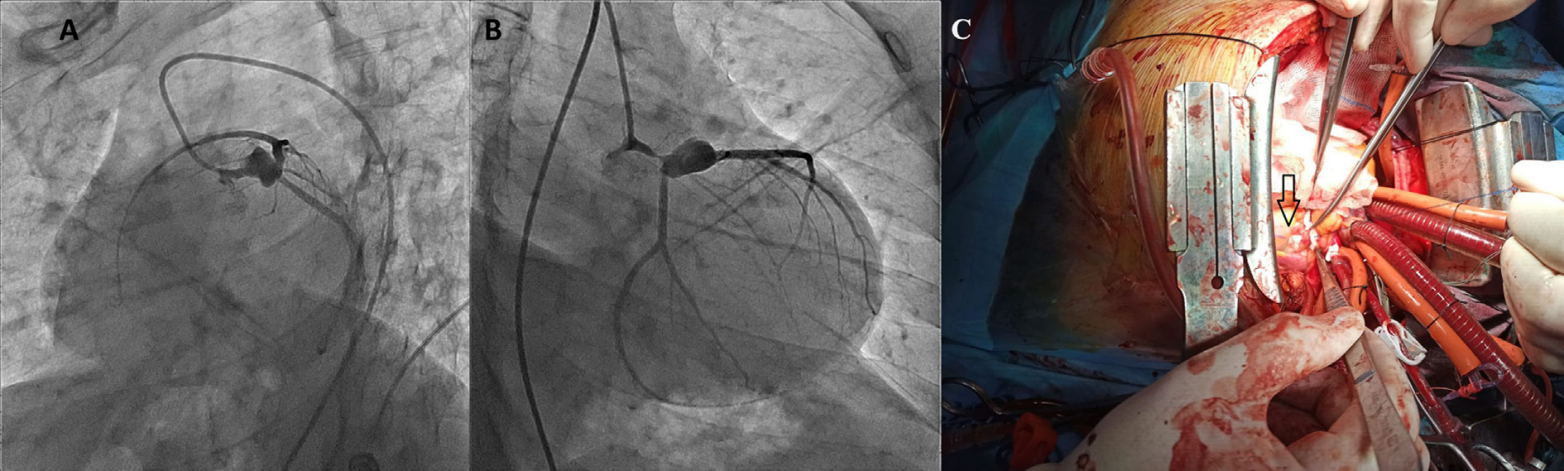
Angiographic image showing aneurysm on spider (A) and left coronary (B) views, Intraoperative image of aneurysm (arrow) (C).

CAA is defined as dilatation of the coronary artery at least 1.5-times the adjacent reference diameter of normal vessel segments on angiography. The exact mechanisms responsible for aneursym formation are not fully understood. Coronary aneurysm formation after stenting can be associated with high pressure inflations, residual microdissection, deep arterial wall injury, coronary stent infection and hypersensitivity reactions to the drug or polymer [2]. The other mechanisms include incomplete healing secondary to the antiproliferative action of the eluted drug and inflammatory changes of the medial arterial wall. Incomplete endothelialisation, which has not been reported after bare metal stent implantation, may have a causative role in aneurysm formation after DES placement as shown in autopsy studies [1], [3]. In our case, aneurysm could be arised from aggressive postdilatation with the increased local drug delivery. There was no flow limiting dissection after stenting. Because aneurysm formation is usually localized to stented segment of the vessel wall, the involvement of the left main coronary artery was the most interesting finding of this case. It would be useful to use optical coherence tomography or intravascular ultrasound for further delineation of aneurysm topography or possible etiology but these imaging modalities are not available in our hospital.

The management of patients with CAA is controversial. Several interventional treatments such as stent grafts or coils have been proposed but a treatment strategy has not been established for this rare clinical entity [4]. Conservative management remains an op-tion for asymptomatic aneurysms. Although stenting of the aneurysms with “covered” bare metal stents has also been explored with good results, several multicentre randomized trials have shown that these stents do not improve clinical outcomes and may be associated with a higher incidence of restenosis and early thrombosis [5], [6]. In the present case, we decided for surgical intervention because of the size and expanding tendency of the CAA presenting as ACS despite dual antiplatelet therapy.

In a previous report, Luthra et al. [7] applied an autologous pericardial patch and BioGlue (CryoLife Inc, Kennesaw, GA) and performed coronary artery bypass in a case of CAA developed 4 years after DES placement. Patch application may be useful to avoid disruption of the native coronary flow but it may enforce aneurysm outside. Also the patch enforcement leaves the potential source of coronary thromboembolism, which is believed to be more prevalent than rupture.

In DES-related CAAs coronary artery bypass graft surgery with ligation or plication has been recommended as a treatment strategy [8], however, it should be noted that this approach may be challenging in cases of proximal LAD artery involvement because this location lies in close proximity to the dorsal portion of the main pulmonary artery. In addition, ligation or plication may be a challenging barrier in performing PCI from the native coronary artery if graft occlusion occurs in the future.

Coronary aneurysm formation is a rare complication of DESs and treatment strategy should be tailored on the basis of the aneurysm size, expansion history, pathophysiology and symptoms of the patient. Surgical approach is seems to be safer and more reliable for repair of a coronary aneurysm devoloping after coronary intervention.

## Declaration of Competing Interest

The authors declare that they have no known competing financial interests or personal relationships that could have appeared to influence the work reported in this paper.
